# Engaging Older Adults to Guide the Development of Passive Home Health Monitoring to Support Aging in Place

**DOI:** 10.3390/s25247413

**Published:** 2025-12-05

**Authors:** Elinor Randi Schoenfeld, Tracy Trimboli, Kaylyn Schwartz, Givenchy Ayisi-Boahene, Patricia Bruckenthal, Erez Zadok, Shelley Horwitz, Fan Ye

**Affiliations:** 1 Division of Population Health and Community Medicine, Department of Family, Population and Preventive Medicine, Renaissance School of Medicine, Stony Brook University, Stony Brook, NY 11794-8036, USA; 2 School of Nursing, Stony Brook University, Stony Brook, NY 11794, USA; tracy.trimboli@stonybrook.edu (T.T.); kaylyn.schwartz@stonybrook.edu (K.S.); patricia.bruckenthal@stonybrook.edu (P.B.); 3 Division of Epidemiology and Biostatistics, Department of Family, Population and Preventive Medicine, Renaissance School of Medicine, Stony Brook University, Stony Brook, NY 11794-8036, USA; 4 Department of Computer Science, School of Engineering and Applied Sciences, Stony Brook University, Stony Brook, NY 11794, USA; ezk@fsl.cs.sunysb.edu; 5 School of Social Welfare, Stony Brook University, Stony Brook, NY 11794, USA; shelley.horwitz@stonybrook.edu; 6 Department of Electrical and Computer Engineering, School of Engineering and Applied Sciences, Stony Brook University, Stony Brook, NY 11794, USA; fan.ye@stonybrook.edu

**Keywords:** sensors, aging in place, smart homes, community engagement, passive remote monitoring, technology acceptance, older adults, health monitoring

## Abstract

**Highlights:**

**What are the main findings?**

- Older adults have an interest in and willingness to have home installed image free sensors in their homes to monitor health. Their willingness was amplified with changes to their living arrangements, health status, and experience with others having a health event that required getting help.
- Newly retired participants were generally younger and more frequent technology users. Thus, they were more knowledgeable about and accepting of incorporating our proposed sensors in their homes.

**What are the implication of the main findings?**

- The study underscores the importance of involving potential users in technology development to create effective and acceptable solutions for aging in place.
- To be accepted, home health monitoring systems must be cost-conscious, privacy preserving and flexible enough to accommodate individuals at different life phases and comfort levels, with different home environments and support systems.

**Abstract:**

By 2050, most adults aged 65 and older in the United States will want to age independently at home, a goal that will strain healthcare resources. Adults aged 50 and older (N = 112) were recruited for study participation between 2018 and 2022. They completed surveys and participated in discussion sessions to explore their needs and opinions regarding smart home sensors. Survey results indicated that older adults’ comfort with smart home sensors increased with their perceived need for monitoring when home alone (OR = 1.46; *p* = 0.012) or sick/recovering from an illness (OR = 2.21; *p* < 0.001). When sick compared to when healthy, individuals were 2.65 times more likely to prefer installing multiple sensors in the living room, 1.75 times more likely in the kitchen, 3.66 times more likely in the bedroom, and 3.41 times more likely in the bathroom (*p* < 0.05). Regarding data sharing, participants were most willing to share information with healthcare providers and family members on a regular basis (80 and 81%, respectively) and 71% on a regular basis or when sick/recovering. Comfort with data sharing with professional caregivers (OR = 1.67; *p* = 0.0017) and monitoring companies (OR = 1.34; *p* = 0.030) significantly increased when sick/recovering. Discussion sessions highlighted overwhelming concerns about personal security/privacy, loss of independence, and ethical issues in data collection. Participants emphasized the need for new systems to be flexible, cost-effective, user-friendly, and respectful of user autonomy, accommodating diverse life stages, comfort levels, home environments, income levels, and support structures. Insights are now informing sensor data collection in our model home. Study findings underscore the importance of involving potential users in technology development to create effective and acceptable solutions for aging in place.

## 1. Introduction

The United States (US) population is undergoing a significant demographic shift, with a rapidly increasing number of older adults. Currently, 17.3% of the US population is 65 and older (57.8 million) [1,2]. By 2050, this figure will grow to 23% of the US population (82 million), with increasing diversity by race and ethnicity. More than ¾ of older adults want to remain in their homes and communities for as long as possible [3,4]. This trend presents opportunities and challenges for healthcare, support services, and home health monitoring for this growing community-dwelling older adult population. While the older US population is growing, the healthcare workforce needed to support older adults is shrinking [5,6,7,8]. Thus, a role exists to further develop and utilize in-home sensors combined with artificial intelligence (AI) to create innovative living environments that support aging in place. These technologies range from passive, ambient sensors that do not require any physical or cognitive burden on users (e.g., radios) to monitor daily activities to wearable devices that track vital signs and detect falls [9,10,11,12,13,14,15,16,17]. With advancing technologies, personal and in-home medical emergency response systems, smart home security, and smart home devices are becoming more sophisticated and accepted [3]. By combining these systems with AI algorithms, one can analyze the generated data to learn an individual’s routines and detect subtle changes in behavior. These data may indicate a health issue, cognitive decline, or an emergency [18]. For example, decreased kitchen activity might suggest a loss of appetite, while changes in sleep patterns could indicate a developing health issue. By identifying these patterns, AI-powered systems can alert caregivers or healthcare providers, enabling early intervention and preventing more serious outcomes.

Prior studies have identified a number of barriers to technology adoption, including: 1—resistance to divulging home difficulties to family members or healthcare providers for fear that they will have to give up their independence and leave their homes [19,20,21,22]; 2—struggling with new technology, especially with increasing age and cognitive decline [23,24,25]; 3—fear of the high cost of purchasing and maintaining required devices and services [24,26,27,28,29,30,31]; 4—feeling that current technology is not designed for users across the lifespan [14,24,30,32]. Despite these barriers, older US-based adults are growing more digitally connected [33]. COVID-19 isolation helped accelerate technology adoption, and recognition of the benefits of using technology to assist with detecting and monitoring illness and increasing age [24]. While 60% of older adults living in the US are aware of in-home sensors, fewer than 5% have adopted the use of these devices in their homes. They either use non-technology methods or are unaware of technology that may be available to support their health [24]. This trend is also observed in Canada. Although passive remote monitoring to support aging in place is publicly funded, utilization is low due to technology maturity and limited program awareness [34].

As technologies to support aging in place become more mature and user-friendly, attitudes and acceptance are slowly changing [3,9,35]. Our study aimed to identify the uses for, acceptance of, challenges, and barriers to adopting passive remote home monitoring to support aging in place, and how the attitudes and acceptance have changed. The primary objectives were to gain insights from community dwelling older adults’ technology use and acceptance, data sharing preferences, opinions on home sensor placement, demographic differences in opinions, and interest in future technologies. We will then utilize knowledge gained to facilitate sensor development and deployment, addressing the needs and challenges of technology adoption to support aging in place.

## 2. Materials and Methods

The study employed a mixed-methods approach, combining surveys and discussion sessions with older adults to inform the development of novel ultrawideband wireless sensors for capturing physiologic and location data, thereby supporting aging in place. These measurements, combined with machine learning and AI-developed algorithms, have the potential to provide greater insights into changes in health among older adults aging in place. To ensure the future adoption of these new devices by community dwelling older adults and individuals with chronic conditions, we are engaging multiple consumer groups throughout the technology development and implementation process to facilitate their integration into home health monitoring activities. Community-dwelling older adults aged 50 and above from the New York metropolitan region within the United States were recruited through partnerships with community organizations and senior living communities. Recruitment methods included invited speaking engagements where the study team presented the study but did not collect identifiable information (using an unsigned consent form). Additionally, recruitment announcements were distributed by community partner organizations, and interested individuals contacted the study team, leading to the completion of signed consent forms or e-consent, where participant identification was known. To be eligible, participants had to be at least 50 years old, live independently in the community, be fluent in English, and provide their own consent. Individuals under 50, non-English speaking, or living in assisted living facilities or nursing homes were not eligible. Since our goal for technology development is to help older adults live in their own homes for as long as possible, we limited participation to community-dwelling older residents who did not require continuous care, as provided by assisted living or nursing homes.

To ensure the protection of human subjects/study participants, Institutional Review Board (IRB) approval was initially obtained in 2018 from the Stony Brook University-wide IRB prior to the start of recruitment and data collection (IRB #878261). IRB review and approval of the study protocol and data collection tools are required to ensure the protection of human subjects throughout study conduct. Subsequent amendments were submitted to accommodate changes in recruitment and data collection settings (in-person, virtual, or hybrid) throughout the study. Data collection involved completing surveys (in-person or online) and participating in scripted discussion sessions offered in-person or via Zoom. Details about survey questions are summarized below. A copy of the full survey is available in Supplementary Materials S1. To help overcome barriers to in-person involvement, the study team offered sessions during daylight hours at locations familiar to participants and accessible to people with disabilities. We sought locations that were easily accessible on foot or by public transportation and provided parking at no cost to participants when applicable. Online sessions provided greater flexibility in scheduling study participation. The limiting factor for participation during COVID-19 was potential participants’ access to the internet and Zoom (Zoom Video Communications, San Jose, CA, USA). Discussion sessions continued until knowledge saturation was achieved.

All survey questions were captured as closed-ended categorical questions. The survey included demographic questions related to age, gender, education, and living arrangements. Age was grouped into 10-year intervals to protect participant privacy and reduce reluctance to disclose age. Given the limited sample size, education was grouped into less than college and college graduate, and living arrangements were recoded into living alone, living with someone, or living in a group setting. The survey included questions about home technology use and comfort with technology to gauge an individual’s potential acceptance of home sensor installation. With respect to acceptance of home sensor installation, participants were asked “how comfortable would you be to have contactless sensors installed in your home to monitor your health and wellbeing in general, home alone, sick, or have a health problem that required continuous monitoring”. The choices were not comfortable at all, a single sensor in some rooms, multiple sensors in some rooms, and comfortable having as many as you want in as many rooms as you want. We then asked participants, “If you had contactless room-based sensors installed in your home, with whom and under what conditions would you be comfortable sharing the information generated from these sensors on a regular basis, and when you are sick or recovering from an illness that may demand immediate attention”. We inquired about their comfort sharing with health care providers (doctors, nurses, social workers), professional caregivers, a company that provides monitoring services, family, friends, and neighbors. The final question regarding sensors installed in the home asked about their comfort and the number of sensors by location in the home. We asked, “In thinking about sensors as a tool to help you when you are either healthy or sick, in which locations would you be comfortable having them installed, living room, bedroom, bathroom, or kitchen?” For each location, we asked whether they would be comfortable with one sensor or multiple sensors when healthy, sick, or recovering. The survey took about 15 min to complete. Each discussion session lasted 1.5–2 h. Sessions began with obtaining or confirming informed consent and using a teach-back method to ensure participant understanding. As part of the session introductions, participants were asked to use only their first names for privacy, as the sessions were recorded. A project overview, goals, and a vignette/persona were presented to facilitate discussion about challenges related to aging in place, followed by questions about personal experiences with home and personal technology use. The vignette/persona highlighted the complexities of aging in place, family dynamics, and the challenges of meeting the needs of an older adult and their family. Sensor and security discussions included a summary of home-based sensor roles and an overview of data collection and security, led by project team members (FY and EZ). A copy of the discussion session presentation and questions posed to participants can be found in Supplementary Materials S2. Participants discussed their comfort levels with sensors, data sharing preferences (who, when, under what conditions), and explored privacy, security, ethical, and societal issues. Participants were encouraged to ask general questions about the study throughout the session. Demographic data, experiences using technologies, the number of home sensors, locations, and with whom and under what conditions they would share data were all collected via a self-completed survey, then analyzed with ages grouped categorically to preserve confidentiality. Study participants did not receive any monetary compensation for participation.

REDCap (Research Electronic Data Capture, Vanderbilt University, KY, USA), a HIPAA-compliant data management system, was designed and used for e-consenting, online survey data collection, and data entry of forms completed on paper. Discussion sessions were recorded using Zoom or a voice recorder, and transcripts were subsequently generated. A scribe was present as a backup at each discussion session. All study session transcripts were reviewed and deidentified as needed to remove names, references to specific locations, and references to healthcare providers before the study team conducted analyses. All recordings and transcripts were stored in a secure, HIPAA-compliant, on-premises web-based tool that offered password protection and role-based file storage, maintained by the University’s Research IT group. Only the grant and study PIs (FY and ERS, respectively) had access to the recordings and transcripts before de-identification. One study team member reviewed and de-identified all transcripts before sharing with the study team for analysis (ERS). Because of the methods for survey completion and discussion group recordings, the study was unable to link survey responses to statements made by study participants during discussion sessions, thus protecting their privacy.

Survey data were analyzed using IBM SPSS (version 29.0.0.0, IBM, NY, USA). Crosstabs and chi-square tests for significance were used for univariate analysis. Logistic regression was used to examine preferences regarding comfort with sensors in the home, sensor location, and with whom to share data generated by the study sensors, with *p*-values, odds ratios (ORs), and confidence intervals (CIs). The logistic models controlled for age, gender, home environment, education, and home living arrangements. Statistical significance in this study was defined as a *p*-value < 0.05.

Data analysis of qualitative data from discussion sessions employed an inductive thematic analysis approach [13,33,34] involving a core multidisciplinary team comprising nursing (KS), geriatrics (TT), epidemiology (ERS), and community collaboration (GA). Team members independently reviewed transcripts, identified themes/subthemes, and then the team collectively reached consensus. Findings were then shared with the full multidisciplinary team (including social welfare, engineering, and computer science) to review results and to guide technology development. A thematic table summarizing the major themes and subthemes was generated for presentation.

## 3. Results

Study participants were recruited from four unaffiliated organizations that support community-dwelling older adults. These included 1—an urban housing complex designed specifically for adults aged 55 and older living independently in the community (55+ community); 2—two suburban university-affiliated organizations that provided programs for community-dwelling older adults (general community); and 3—an independent living division from a rural continuous care community (continuous care). The leadership from the 55+ and the continuous care communities recruited and invited participants to the in-person sessions and coordinated all in-person activities. No names or contact information were shared for these participants. Thus, these participants remained anonymous to the study team. The university-affiliated organizations sent recruitment emails to their members, providing instructions to visit the REDCap survey site or contact a study member (ERS), who would coordinate sessions directly with participants. Discussion sessions held in the 55+ community and the independent living section of the continuous care facility were in-person. Discussion sessions conducted with local community-dwelling residents began as inperson and moved to fully online due to COVID-19.

### 3.1. Survey Results

#### 3.1.1. Demographics (Table 1 and Figure 1)

A total of 112 community dwelling older adults were recruited from the general community and older adult focused independent living complexes. These individuals completed the study survey and participated in one of 11 discussion sessions. The older adult focused communities included a 55+ independent housing complex in an urban setting and an independent living complex within a rural continuous care community. In 2018, 24 individuals (21%) were recruited from the 55+ apartment complex. Over a 3-year time period (2019, 2020, 2022), 68 individuals (61%) were recruited from the general community. In 2022, 20 individuals (18%) were recruited from the independent living complex within the continuous care community.

Participants ranged in age from 50 to 90+, with 70–79 year olds making up the largest group of participants (n = 50; 46%); 53% were female; 78% had a college education or higher; and 68% lived with another individual (e.g., spouse, significant other, family member). Overall, participants were technology users, with at least 75% using smartphones, computers, texting, and video conferencing. Over 80% had access to cable and the internet via Wi-Fi (Table 1).

In a logistic regression analysis examining differences in technology use by demographics, participants in the 55+ community were more likely to report basic cell phone use than those recruited from the other two communities (OR = 8.4; *p*-value = 0.0022). Similar findings were seen for participants who reported living in a group setting (OR = 15.2; *p*-value = 0.0073). Participants living in the general community were significantly more likely to have cable in their home (OR = 3.6; *p*-value = 0.036). Female participants were more likely to report using a wearable health device (OR = 7.5; *p*-value = 0.01). No other demographic factors were significantly associated with technology use. Due to the timing of data collection, it was not possible to differentiate between the recruitment site and the impact of COVID-19 on technology use because of collinearity.

**Figure 1:**
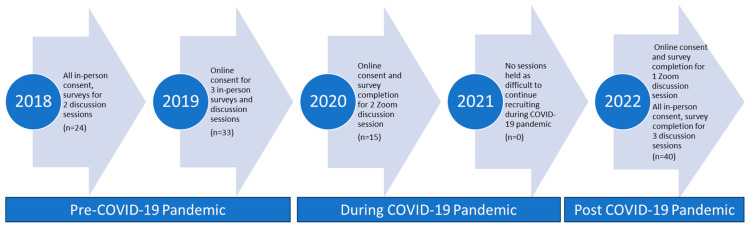
Study timeline and data collection methods.

Surveys were completed in person by 77 participants and online in REDCap by 35 participants. Discussion sessions were initially held in person in 2018 and 2019. We transitioned to Zoom in 2020 and 2022 as the COVID-19 pandemic took hold in the US. As the pandemic dissipated, we resumed in-person sessions in the latter part of 2022. Between 7 and 24 individuals participated in each discussion session. No sessions were held in 2021 due to the COVID-19 pandemic, which limited our ability to recruit participants. The recruitment timeline and data collection methods are shown in Figure 1. Two fully in-person sessions were held in 2018 (n = 24 participants); 3 hybrid sessions were held in 2019, with online consent, surveys, and discussion sessions held in person. In 2020, due to the COVID-19 pandemic, consent, surveys, and 2 discussion sessions were completed via Zoom (n = 15). In the latter part of 2022, as the United States began reopening, we were able to hold 3 fully in-person sessions (n = 20).

**Table 1:** Demographics of survey and discussion group participants.

	(N = 112)
Participant Demographics	n (%)
**Recruitment site**	
55+ apartment complex in an urban setting	24 (21)
General community in a suburban setting	68 (61)
Independent living complex in a rural continuous care community	20 (18)
**Age**	
50–59	1 (1)
60–69	26 (24)
70–79	50 (46)
80+	32 (29)
missing	3
**Female Gender**	57 (53)
**Education**	
<College	24 (22)
College graduate missing	83 (78) 5
**Living arrangements**	
Live alone	20 (19)
Live with someone	73 (68)
Live in a group setting missing	14 (13) 5
**Home technology use**	n (%)
**Have technology access at home**	
Basic cellphone (e.g., flip phone)	48 (54)
Smartphone (e.g., Apple iPhone, Samsung Galaxy)	93 (89)
Tablet (e.g., iPad, Samsung)	67 (67)
Computer (desktop or laptop)	88 (85)
Cable/satellite access (e.g., Spectrum, Verizon)	88 (87)
Text messaging on your cellphone	96 (92)
Video conferencing (e.g., Zoom)	78 (75)
Internet access via WiFi (wireless)	93 (89)
Home health monitoring (e.g., BP machine) (n = 44) *	28 (64)
Wearable health devices (e.g., fitbit, apple watch) (n = 42) *	13 (31)
Falls detection monitoring *	6 (14)
**Very comfortable with technology use**	
Making or receiving a call on your cellphone	90 (88)
Using your tablet (e.g., iPad, Samsung)	32 (71)
Using your computer (desktop or laptop)	33 (75)
Using an app on smartphone, tablet or computer	34 (74)
Text messaging on your cellphone	40 (87)
Video conferencing	30 (67)
Connecting to the internet	80 (82)
Home health monitoring devices *	22 (48)
Wearable health devices *	15 (33)
Falls detection monitoring *	5 (11)
Telehealth *	11 (26)

* question not asked of all participants.

#### 3.1.2. Sensor Preferences

Early in the study, participants’ overwhelming negative reaction to the potential for sensors to produce any type of body visualization using thermal, depth, or wireless radio sensors influenced our sensor development direction. Participants’ reactions to these sensor types led the engineering team to focus development efforts on ultrawideband (UWB) radio-based sensors, which do not collect or produce any visual information. Consequently, later sessions focused on potential uses and barriers to UWB sensor deployment in the home, rather than other sensor types.

#### 3.1.3. Comfort with Home Installed Sensors by Number of Sensors and Individual Health Status (Figure 2 and Table 2)

**Figure 2:**
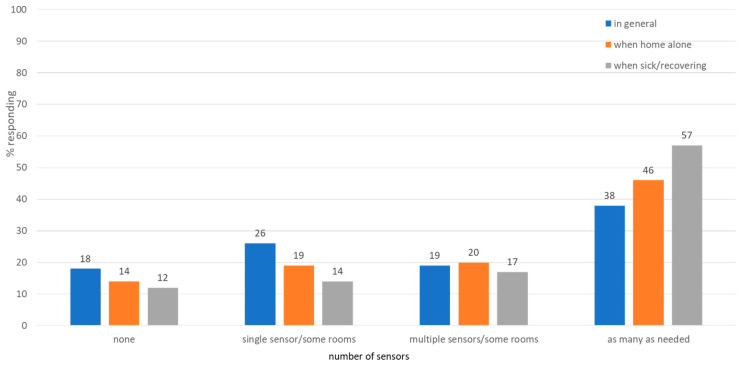
Comfort with home-installed wireless radio sensors by number of sensors installed—in general, when home alone, or when sick or had a health problem that requires continuous monitoring.

We inquired about comfort with home-installed sensors under different health statuses (in general, home alone, sick, or with a chronic condition), and the number of sensors to be installed (none, single sensor, multiple sensors, as many as needed). As shown in Figure 2, a greater percentage of participants were comfortable with having as many sensors as needed installed in the home across all three health statuses. There was a significant difference in the preference for as many sensors as needed by health status. In the event that a participant was sick, they would be 1.5 times more likely to want as many sensors in their home as needed. Compared with the general health scenario, they would be 2.21 times more likely to want as many sensors as needed when sick (Table 2).

**Table 2:** Strength of the association between health status and installation of as many sensors as needed based on logistic regression analysis.

Health Status	Odds Ratio (OR) *	95% Confidence Interval	*p*-Value
Sick vs. Home alone	1.52	1.13–2.04	0.006
Sick vs. in general	2.21	1.52–3.21	<0.001
Home alone vs. in general	1.46	1.09–1.95	0.012

* Based on the logistic regression model controlling for age, gender, home environment, and education.

#### 3.1.4. Comfort with the Location for Home-Installed Sensors by Health Status and Number of Sensors (Figure 3 and Table 3)

**Figure 3:**
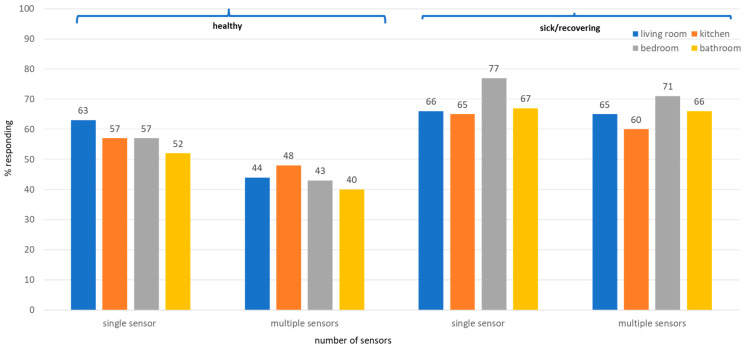
In thinking about sensors as a tool to help you when you are either healthy or sick, in which locations would you be comfortable having them installed?

We inquired about the in-home locations (living room, bedroom, bathroom, kitchen) and the quantity (one sensor vs. multiple sensors) in which participants would be comfortable having sensors installed, whether they are healthy or sick/recovering. Participants were most comfortable having one sensor installed in the living room when they were healthy and multiple sensors when they were sick or recovering (Figure 3). The percentage of participants expressing interest in home-installed sensors increased during sickness or recovery, especially in more private areas (i.e., the bedroom and bathroom). As presented in Table 3, when sick compared to when healthy, individuals were 2.65 times more likely to prefer installing multiple sensors in the living room, 1.75 times more likely in the kitchen, 3.66 times more likely in the bedroom, and 3.41 times more likely in the bathroom (*p* < 0.05). When healthy individuals were significantly less likely to want multiple sensors in any room, which is reflected in the odds ratios (all <1.0). Overall, these data show that individuals were more comfortable with having more sensors installed in their homes when they are sick/recovering, consistent with the use of close patient monitoring during recovery from illness.

**Table 3:** Strength of the association between the location of home-installed sensors by health status and number of sensors using logistic regression analysis.

Location Within the Home	Preference for Single vs. Multiple Sensors	Odds Ratio *	95% Confidence Interval	*p*-Value
**Living room**				
When sick	Multiple vs. single sensor	0.95	0.62–1.46	0.805
When sick vs. healthy	Multiple sensors	2.65	1.67–4.20	<0.0001
When sick vs. healthy	Single sensors	1.14	0.71–1.84	0.591
When healthy	Multiple vs. single sensor	0.41	0.26–0.63	<0.0001
**Kitchen**				
When sick	Multiple vs. single sensor	0.75	0.52–1.08	0.124
When sick vs. healthy	Multiple sensors	1.75	1.12–2.71	0.013
When sick vs. healthy	Single sensors	1.41	0.99–2.01	0.056
When healthy	Multiple vs. single sensor	0.60	0.40–0.92	0.019
**Bedroom**				
When sick	Multiple vs. single sensor	0.62	0.39–0.99	0.047
When sick vs. healthy	Multiple sensors	3.66	2.29–5.84	<0.0001
When sick vs. healthy	Single sensors	3.09	1.85–5.16	<0.0001
When healthy	Multiple vs. single sensor	0.52	0.34–0.79	0.0023
**Bathroom**				
When sick	Multiple vs. single sensor	0.92	0.60–1.42	0.71
When sick vs. healthy	Multiple sensors	3.41	2.22–5.24	<0.0001
When sick vs. healthy	Single sensors	2.09	1.38–3.17	0.0005
When healthy	Multiple vs. single sensor	0.57	0.39–0.83	0.0033

* Based on the logistic regression model controlling for age, gender, home environment, education, and home living arrangements.

#### 3.1.5. Data Sharing (Figure 4 and Table 4)

**Figure 4:**
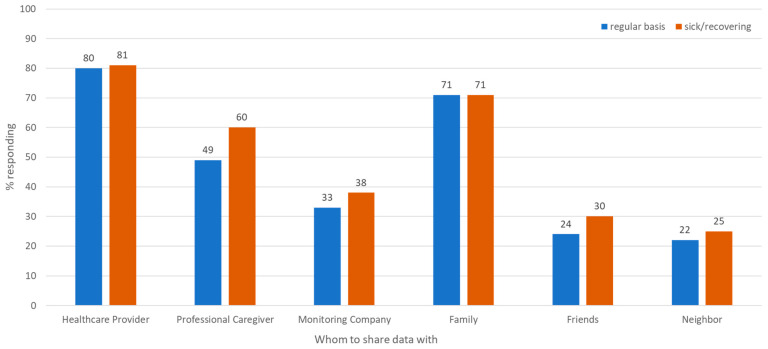
If you had contactless room-based sensors installed in your home, with whom and under what conditions would you be comfortable sharing the information generated from these sensors?

We asked participants with whom they would be comfortable sharing sensor-generated data and under what conditions. Participants were more willing to share their sensor data with a healthcare provider (80% on a regular basis, 81% when sick/recovering) and family member (71% both on a regular basis or when sick/recovering) (Figure 4). Less than 60% were willing to share their data with professional caregivers, monitoring companies, friends, or neighbors on a regular basis or when sick/recovering. There was no significant difference in comfort with sharing data with healthcare providers and family on a regular basis or when sick (Table 4). Participants were significantly more likely to want to share data with Professional Caregivers and a Monitoring Company when they were sick or recovering from an illness than on a regular basis (ORs of 1.67 and 1.34, respectively). No significant differences were observed in data sharing with other groups. These findings reflect greater comfort and trust among healthcare providers and their families with very personal, confidential information related to an individual’s health and well-being. When sick/recovering, individuals are more willing to share their data with others, reflecting possibly a hope that such parties (e.g., professional caregivers, monitoring companies) might provide additional benefits during difficult times.

**Table 4:** Strength of the association between health status and who an individual would be comfortable sharing their sensor data when sick versus on a regular basis from logistic regression analysis.

With Whom to Share Sensor Data	Odds Ratio (OR) *	95% Confidence Interval	*p*-Value
Healthcare Provider	1.00	0.67–1.00	0.989
Professional Caregiver	1.67	1.21–2.30	0.0017
Monitoring Company	1.34	1.03–1.74	0.030
Family	1.00	0.69–1.46	1.00
Friends	1.42	0.98–2.05	0.061
Neighbors	1.23	0.86–1.74	0.256

* Based on the logistic regression model, controlling for age, gender, home environment, and home living arrangements.

### 3.2. Summary of Discussion Session Findings—Themes Identified from the Discussions

Thematic analysis identified 3 major themes and six subthemes. Table 5 provides a thematic table summarizing the themes, subthemes, and key findings from our discussion sessions, reflecting participants’ perspectives on aging in place and home monitoring. In the following sections, we present details for each theme, including relevant participant quotes.

#### 3.2.1. Theme #1: Challenges to Aging in Place

Participants noted physical and technology related challenges to remaining independent and aging in place. Physical challenges included trouble with stairs, inability to reach cabinets, hearing loss, and trouble seeing the phone to dial. Difficulties with technology included the expense of buying and maintaining, the difficulty of use, and the need for assistance with repairs. During the COVID-19 pandemic, challenges included disruptions to daily routines, accessing food, and seeking help with household tasks or broken technology.

Participants expressed concerns about being unable to care for themselves, particularly in an emergency, when living independently and far from their children, other family members, and/or neighbors. During the COVID-19 pandemic, participants were particularly concerned about disruptions to their daily schedules, accessing food, and seeking assistance with household tasks and technology when it was not functioning properly, or they were unsure how to use it. Participants noted that before the COVID-19 pandemic, they were accustomed to going places (e.g., museums, restaurants, senior centers, libraries), meeting with friends, and engaging in “shopping as an activity”. These were all curtailed or stopped during the height of the COVID-19 pandemic. Participants expressed concerns about online shopping, as they preferred seeing and feeling products before making a purchase. Food shopping became one of their biggest challenges during the COVID-19 pandemic. Participants post-COVID-19 were interested in continuing to visit with friends.

Participants discussed various lifestyle and living arrangement adaptations due to health changes, family and friend relocation, loss of loved ones, and retirement from work. Individuals who found themselves now living alone or who were home alone during the day were much more interested in having sensors in their homes to alert others if there was a health “problem.” A few participants mentioned health events that happened to friends of theirs (e.g., stroke, heart attack, fall) that resulted in their review of their own home safety procedures and how to get help in an emergency. These individuals were more interested in having technology to help monitor their health when they were home alone. Those with chronic conditions were more interested in learning about technologies to help them age in place. Newly retired participants who still had more disposable income, were also more technologically connected (e.g., ring doorbells, digital locks) than those who were further from retirement. Thus, they are more accepting of installing other devices in their homes than older participants. Many participants asked if, at some point, insurance might also cover our technology.

#### 3.2.2. Major Theme #2: Home Monitoring Concerns and Questions

Participants were overwhelmingly concerned about privacy and security, and questioned who would see their sensor-generated data and derived information, and how it would be used. They were also worried about the amount of data collected and the burden it could place on themselves, their loved ones, and their healthcare providers. Participants were concerned about the potential impact on their independence if their children knew too much about their activities. There was concern that if the children knew too much, they would “have the talk with them” about no longer being able to live in their own homes. Participants were overwhelmingly concerned about the type of images that the sensors would collect. No one wanted their children to see them without clothes (e.g., in the shower, changing in the bedroom) and did not want their families to know “when they are entertaining”. Participants were adamant about maintaining control over when they would be monitored. They strongly felt they should be able to turn monitoring on and off to protect their privacy and that of visitors (e.g., grandchildren, house guests). They did not want young children monitored in their homes and were concerned about protecting visitors’ privacy from the collection of sensor data.

Participants expressed concerns about the burden of home sensing, specifically the added expense/cost of purchasing and maintaining the technology, especially for those on a fixed income. This concern was a continuation of discussions about the support for currently used technologies when they break, especially the challenges of finding support technicians to help one remain “connected”. Participants noted potential adoption barriers, including cultural differences and disabilities related to aging (e.g., vision and hearing loss, assistive device use). Participants under 70 years of age were more frequent technology users and thus were much more knowledgeable about and accepting of incorporating the proposed sensors into their homes.

#### 3.2.3. Major Theme #3: Caregiver Concerns

Although participants were older adults themselves, they also served as caregivers for spouses, siblings, or other relatives. As caregivers, they reported feeling tired and constantly worried about watching their significant others. Due to disabilities and/or cognitive decline, some significant others wander in and out of the home, placing a burden and pressure on the caregiver. These concerns extended to home safety. For example, the significant other may leave something on the stove, fall, and remain on the floor until someone finds them. Another significant challenge for participants was balancing their own lives as caregivers with the needs of the individuals they care for, such as attending doctor appointments, obtaining food, and visiting with friends. Caregivers felt they needed help monitoring their loved ones while out of the home, such as when running errands. “Knowing everything is ok at home while I am out would give me peace of mind”. They discussed the advantages of monitoring loved ones when they were unavailable. Participants noted that getting in the car and driving 1 h or more to “run” to a relative in an emergency becomes more challenging as they age. Others mentioned that their children had moved out of the region, so having them as emergency contacts was not helpful. A common challenge for study participants and all older adults is when to stay in their own home and when to move. Individuals who moved due to changes in their own or their significant other’s health reported losing their friends and support networks. Thus, they reported the need to develop new support connections.

COVID-19 was also noted to have a significant impact on the older adults they care for. During the height of the COVID-19 pandemic, participants remained in touch with their friends and family via phone and other technologies (e.g., Zoom and FaceTime). Caregivers have reported that they were surprised at how much their relatives’ health declined because of COVID-19 shutdowns and isolation, and how much their homes needed repairs when they finally visited in person. They stated that “much was masked by only seeing my relative from the waist up”.

## 4. Discussion

### 4.1. Principal Discoveries

In total, we recruited 112 community participants who represent our future home-health monitoring users. Using a combination of surveys and discussion sessions, we explored in depth new areas of acceptance and adoption of sensor use for home deployment. Our study appears to be the first to explore sensor configurations by room and health status across different home living arrangements, as well as the conditions under which data is shared and with whom. Study findings have guided technology choices and are guiding our sensor system design. Based on our findings, we are creating a system that is flexible enough to accommodate individuals at different life phases and comfort levels, across varying home environments and support systems, while providing a secure, privacy-preserving environment. Though prior studies have identified older adults’ comfort with and interest in individual components for home sensing, our study is unique in being more comprehensive in exploring the extent of home sensing, data sharing and the need for technology adaptability across the lifespan [31].

Our survey found that participants had different opinions as to the amount and location of sensor deployment, depending on their health status. There was greater comfort with sharing home monitoring data with healthcare providers and family, whether healthy or sick. The extent to which individuals were comfortable sharing their data beyond health care providers and family increased when sick or recovering from an illness though comfort remained far less. From discussion sessions, we further identified that cost, privacy, security, the potential for system hacking, and the availability of a support system (e.g., family, friends, neighbors, professional caregivers) were major concerns. Participants noted the need for assistance with technology related issues after deployment, as reported in prior quantitative research [11,36]. Participants were very enthusiastic about the vision of our proposed sensor system. They felt that having home monitoring as they aged would provide themselves, loved ones, caregivers, and healthcare providers with valuable information to help them remain safe at home and offer insight into their health between healthcare provider visits. Based upon these findings, initial home deployment for home health monitoring may be most successful targeting individuals post hospitalization or those with chronic health conditions. These individuals may be more amenable to home health monitoring to support aging in place.

Participants from 55+ communities and continuous care facilities were more comfortable with monitoring, as these settings already have in-room help cords and wearable alert systems in place for residents. Although participants noted their availability, it is not always acceptable or feasible. This suggests that individuals already engaged in continuous care arrangements are highly receptive to data sharing, likely viewing it as integral to their well-being and health management. Experiences with participants’ own or others’ changing health status helped participants appreciate how continuous home sensing could assist them in getting help when needed and in aging in place. COVID-19 has altered the perspective of older adults and their caregivers on the use of technology. Participants noted 1—increased use of telehealth to connect with their medical care team; 2—use of Zoom and FaceTime to overcome social isolation and stay connected, and willingness to continue to use these technologies; and 3—increased acceptance of smartwatches, especially the Apple Watch, as it now includes a falls-detection alert system. Survey findings and discussion sessions reveal shifting attitudes among participants regarding home monitoring, particularly in relation to changes in health status, home environment, and the absence of onsite caregivers. While living rooms and kitchens generally showed high comfort, comfort in bedrooms and, particularly, bathrooms was lower across different home environments and health conditions, highlighting privacy concerns or perceived intrusiveness in more private spaces, which was further supported during discussions. However, for those facing declining health or requiring additional care, even bathrooms can offer high comfort with multiple sensors, indicating that the perceived need outweighs privacy concerns.

The combined findings from our survey and discussion sessions are driving our sensor kit creation and home deployment. We envision developing a sensor kit that is inexpensive, self-contained, and remotely monitorable. Based on initial participant input, we have focused on using off-the-shelf ultra-wideband (UWB) sensors that do not generate any body images to address privacy concerns while remaining cost-effective for widespread deployment. We are pairing this UWB sensor with a Raspberry Pi for data collection, with data sent initially to an edge machine and then to a secure Cloud. All signal processing, sensing data extraction algorithms and code are custom-developed and reconfigurable to meet the varying needs of older adults aging in place. We have deployed 16 sensor kits in a campus-supported model home, where we are collecting participant data as they complete a series of scripted activities of daily living [16].

Another strength of our study is the multidisciplinary team approach, which includes academics, healthcare providers, community leaders, and older adults who collectively participate in discussions throughout the technology development life cycle. This approach can positively impact future technology adoption [37]. Our research methods for engaging older adults align with prior work that explored the acceptance of technology among older adults. The strength of using a persona developed by community collaborators to guide discussions on technology development was supported by prior research [38,39]. Dupreez et al. employed community engagement methods to develop a persona that reflected the local community. This persona was then utilized to inform technology design, providing a human-centered approach through an iterative design methodology [38]. Tiersen et al. employed a participatory approach to understand the priorities of individuals living with dementia and their caregivers, and to inform the design and implementation of technologies that support these individuals’ functional and psychological needs [39]. Similarly, including the opinions of multiple user groups in our study helped prioritize technology development components.

Many prior studies did not employ community engagement in technology design; instead, they used a standard development, testing, and implementation format that did not include input from the potential user community until the technology was ready for testing or product rollout [40,41,42,43,44,45,46]. Only a few studies have used surveys to explore the concerns, needs, and interests of older adults regarding technology use, home health monitoring and the association with aging in place [30,36,47,48,49,50]. Each year, AARP conducts surveys of older adults about their current technology use. According to the 2025 AARP survey, a greater percentage of older adults are embracing technologies to facilitate aging in place and appreciate those that monitor and support their health. The needs of older adults partially contributed to this increase in technology acceptance during the COVID-19 pandemic, as they were more socially isolated [30,35]. These findings align with this study’s results, showing increases in technology use post-COVID (e.g., smartphones and Zoom). A national study in South Korea found that participants aged 75 and older were more likely to desire to age in place. Individuals with higher education and higher income were also more likely to age in place [50]. These findings help support the observations in our study. Though our participants are self-selected, their demographics are similar to those in the South Korean study by age, education, and economic status. The study participant demographics reflect the region of the US in which our study was conducted. Thus, we have confidence that the findings presented here reflect the broader voice of older adults aging in place in our region and can guide the development of new technologies to facilitate aging in place.

### 4.2. Comparison with Prior Work

Our findings are consistent with prior research that identified the perceived value of health monitoring technology [9,35,36,51,52,53,54,55,56], privacy/data sharing concerns, and data ownership [32,47,56,57,58,59,60,61,62], technology use confidence, and the burden of use [32,51,54,55,60,63,64,65,66], system flexibility [56,67], increased acceptance with age/frailty [43,49,68,69], or when it can help older adults live longer at home [51,70]. Prior work examined the role of home monitoring for activities of daily living/instrumental activities of daily living, sleep, mobility, falls, location tracking, and abnormal behaviors [35,71]. Similarly to other studies, older adults appeared accepting of the technologies as long as the cost was reasonable, benefits outweighed the concerns about use/data collected, privacy was preserved, the user maintained control over the technology, and access to the generated data to preserve independence [42,43,56,72,73]. Our study, along with others, supports the need for engagement with a community and potential users throughout the technology development lifecycle, thereby continuing to address user needs and strengthening our potential for successful adoption [32].

#### 4.2.1. Theme #1-Challenges to Aging in Place

Accepting and using technologies to support aging in place was a major theme across our study. The strongest predictors of new technology adoption by older adults included the value of the technology, the impact of technology use on quality of life, and confidence in using the technology [51,74]. Resilient individuals are also more likely to adopt new technologies before they are needed, and as health declines [52]. Our participants who learned about friends’ new adverse health events (e.g., heart attack, stroke) were more interested in adopting new technologies to detect health events or provide assistance earlier when needed.

Our discussion group participants were more accepting of home monitoring following the COVID-19 pandemic. Studies conducted in Canada and the United States noted similar findings, with participants expressing their desire to continue using the technology they adopted during the COVID-19 pandemic [53,63]. Results from a Singaporean study conducted during COVID-19 found that digital technology acceptance had shifted from pre-COVID, with perceived benefits including ease of use, social influence, and confidence in use [55]. This further supports our participant observations and those from other studies that learning new technologies is more challenging without external support, education on use, and increased complexity [51,71]. In our findings, a participant stated “Newer technologies are not made for me but rather designed for younger people,” a sentiment supported by the 2024 AARP survey [24]. This further confirms the need for engagement throughout the technology development lifecycle to overcome barriers, educate, and allay fears of technology use and adoption.

Another avenue to facilitate adoption identified in our discussion sessions is support from the older adults’ families, extended families, friends, and neighbors. During the height of the COVID-19 pandemic, technology failures posed a significant challenge to its use. Without their support system, older adults found it difficult to maintain their technologies when they “broke” and to learn new technology on their own (e.g., Zoom). Though conducted pre-COVID-19, the importance of family in supporting older adults’ technology adoption and use was identified by Dickman Portz [75]. As noted by our study and others, the widespread adoption of Zoom during the height of the COVID-19 pandemic provided a tool to stay connected with family and friends. There was overwhelming agreement that they would continue to use Zoom to “stay connected” beyond the pandemic [53,63].

Participants expressed concerns about the cost of purchasing and supporting new technology, particularly when expendable income may decrease, and about accepting the trade-off between these expenses and the benefits of remaining in a familiar and comfortable environment. These concerns were similarly raised in studies conducted both inside and outside the United States [24,52,63]. Generational differences for monitoring acceptance identified in our study were documented in previous studies where those < 70 years of age [65,71] and those pre-retirement were more enthusiastic about accepting and using home environmental sensing [76].

#### 4.2.2. Theme #2-Home Monitoring Concerns and Questions

A burgeoning field of research concerns the ethics, security, privacy, and trust associated with the expanding use of smart home technology [32,45,52,56,60,73,77]. The fear of data collection without consent and illegal data access ranked as top concerns by others [78,79]. There is a misconception that the aging population will readily accept medical technology due to health concerns, without a deeper understanding of who receives the information and how the data is presented [78,80]. Participants in our study were very concerned about who they would feel comfortable sharing sensor data with, under what conditions, and who in the home they would include in data collection. As more smart home technologies have entered the market, a dynamic tension has emerged between the benefits of monitoring and concerns about privacy, data breaches, and an affront to autonomy among older adults [48,60,81]. Prior investigations have identified fears of asking for help as a barrier to technology adoption, due to concerns that others would perceive them as no longer able to perform tasks, which can translate into a burden on others and a loss of control [59,80]. These findings offer insights into the concerns our participants expressed about aging in place and the reluctance to share sensor data, which could lead to questioning one’s independence. Crotty et al. found differences in perspectives between caregivers and older adults regarding what constitutes “data burden”. Older adults did not want to feel “spied on” or “lose control” over decisions if data were shared. The study concluded that there is not a “one size fits all” model for data sharing among older adults and those who support them. This concept was included in all our discussion sessions and influenced how we are designing our sensor kit to offer options and accommodate individuals at different stages of aging with varying opinions and comfort levels regarding data sharing. As highlighted in our discussions, these concerns are more significant among older participants, as monitoring can impact their independence and autonomy. Dermody et al. found that their participants initially had very negative perceptions about home monitoring, but these perceptions changed as they learned more from discussions with investigators. Participants were generally uncomfortable using cameras and having their audio recorded [48]. Early in our study, participants expressed strong concerns about using visual data (including coarse-grained depth cameras), prompting our technology development team to focus on ultra-wideband sensors that use radio waves and produce no visual data. In the Dermody study, participants were also asked about their sensors’ ability to detect family pets [48]. Our participants took these concerns one step further, asking that our system be able to be turned on and off to control who and when individuals are monitored (e.g., house guests, grandchildren).

Individuals with the most and those with the least technological experience were more concerned about privacy and data sharing. Tech-savvy individuals were concerned about their existing knowledge base, while tech novices were concerned about the unknown. Prior studies support findings about this latter group [48,65,72]. The middle group was generally younger, adopted technology for themselves and their homes, and welcomed the inclusion of our technology to support aging. Similar trends were found in the United Kingdom [71]. Prior research has identified that with education and improved knowledge, older adults are more likely to be accepting of technologies that support aging in place and health monitoring. They noted a balance between giving up some privacy to maintain independence and obtaining support when needed [72].

The burden on family and friends resulting from data overload was a recurring theme in our study and prior research with older adults [48,59,65]. In focus groups conducted at Tiger Place, a senior living community in the US state of Missouri, they assessed interest in and concerns about home monitoring. Participants felt there needed to be a balance between safety and privacy, and understanding the benefits of monitoring [82,83,84]. Healthcare providers and technology developers have also raised concerns about the role of smart device data in healthcare, its misuse, burden on the healthcare system, data ownership, and the lack of smart device data standards as a limitation to its adoption [56,60,61]. Our participants’ concerns mirror concerns about the burden on individuals and healthcare providers to address the health changes identified, as well as about how the data would be used, which could infringe on privacy. To overcome these privacy and security challenges as described here, all potential users/technology consumers (e.g., business, academia, medicine, health insurance, providers, and users) must come together to speak to the challenges and benefits of such data collection while preserving the privacy and security of the individuals and the data that they generate.

#### 4.2.3. Theme #3 Caregiver Concerns

Prior studies have explored older adults’ readiness to adopt smart home technologies. Previous research has shown that having a smart home provides “eyes on” support to relieve caregivers’ burdens and gives older adults confidence that continuous assistance is available [48,85]. This becomes even more important with long-distance caregiving, as noted in a systematic review of attitudes toward technology use by distant caregivers [81]. Many of our study participants noted their role in supporting family members with decreased mobility and/or cognitive decline. These participants were enthusiastic about the potential of our sensors to provide comfort when they could not physically be with their loved ones, which was especially challenging during and immediately following the height of the COVID-19 pandemic. Participants further noted their own need to leave home to do work, run errands, and make doctor appointments, among other things, and not have the peace of mind of knowing that “everything is ok at home”.

Though prior studies mentioned cameras [85], newer technologies, like those we are developing, overcome older adults’ privacy concerns while still addressing caregivers’ concerns. A mixed-methods study by Piau et al. determined that caregivers were very enthusiastic about using a smart home to monitor older adults for falls, medication use, and changes in functioning. This finding adds to the uses identified by our respondents for smart technology’s role in supporting aging in place [79].

Similarly to our findings, other studies noted the benefits of health monitoring in aiding medical care [65,71]. Younger participants in the Camp study noted the same trend as ours, with caregivers identifying the advantages of home monitoring for health status and well-being (e.g., fall detection) for older adults they care for [71]. In contrast, Egan et al. found that caregivers lacked confidence in the currently available technology to support caregiving, citing issues such as irrelevance (targeted towards younger individuals), cost, lack of support, and the technologies not meeting their needs [86]. Many of these challenges were identified in our discussions and continue to guide our technology development and testing. Our participants noted the challenges of convincing the older adults they care for to embrace monitoring technologies and how long it takes for all involved to agree. This trend was highlighted in a study by Thilo et al., who described the adverse reactions of older adults to recommendations for home monitoring devices and the need to balance monitoring with independence [87].

### 4.3. Limitations

The limitations of the study fall into three categories: participant demographics, methodological execution, and the scope of the technology investigated. The study faced demographic limitations, primarily in terms of educational and race/ethnicity diversity, which were attributed to the specific region where the research was conducted. The research was also confined to English-speaking participants due to the demographics of the local residents. The necessary transition to fully online recruitment and Zoom discussion sessions during the COVID-19 pandemic may have introduced sampling bias, potentially limiting the participant pool to those already possessing access to computers and the internet, thereby restricting applicability to communities with less computer and internet access, particularly older adults aged 80 and older. Despite these shortcomings, participant representation was balanced by sex, age, and home companionship. We plan to expand outreach beyond the local region and to diverse communities to address these demographic limitations in future work. Even with these limitations and COVID-19 adaptations, we may have identified the individuals who would become early adopters of our technology and thus those we should target to facilitate adoption.

Methodological restrictions included data linkage and sample size. To preserve participant confidentiality, the study team chose not to link individual survey responses with the individual participant’s discussion session data. Though a standard methodology, it limits the ability to conduct analyses and sub-analyses exploring the associations between specific demographics (such as age or living arrangements) and the themes identified in the discussion groups. Additionally, the relatively small survey sample limited the ability to perform complex sub-analyses and to examine interactions in the survey data. An early study challenge was engaging in a discussion of technology use with an audience that lacked a frame of reference for the novel technologies we were exploring; this was resolved when the study team developed and used a vignette/persona to help participants understand the technology’s potential uses by first discussing a fictional individual.

A limitation of the investigation’s scope was the decision to focus exclusively on contactless, home-installed sensors, without discussing wearable devices. Investigators chose this approach to eliminate the inherent burden of charging, wearing, and potential breakage associated with wearables, as well as long-term compliance challenges based on individual demographics, stage of life, device usability, etc. [88,89]. This specific focus on home-installed sensors was deemed appropriate because older individuals, especially those with worsening chronic conditions, tend to spend extended periods in their homes, making in-home monitoring a logical choice to support aging in place, as noted in prior studies by our team and others [71].

### 4.4. Relevance to Future Technology Design and Adoption for Use by Older Adults

Study findings continue to guide us as we build, test, and deploy our sensor kits in the community. The decisions made in choosing the sensors to use, identifying the chronic conditions that are important to older adults and healthcare providers for predictive analytics development, and the community and provider groups we are working with are all direct results of this study’s findings. We believe that through community engagement and discovery, researchers can develop stronger ties with future users and learn together what will and will not work to bring technology to market. History shows that technology developers who do not involve potential users throughout development and deployment tend to produce technologies that lack widespread adoption. The methods we have chosen enable us to learn from and work with our target communities, which will help ease our next steps in home testing and deployment.

The knowledge gained from this study has applicability to any technology development for older adults. The overarching theme from this study is that a one-size-fits-all technology to support aging in place will not be embraced. The technology must be easy enough for the older adult to use, non-burdensome, affordable, shareable with others, privacy-preserving, and provide peace of mind for caregivers. The major takeaway is that as individuals experience different health concerns with age, their aging-in-place needs and the people involved in their care will change. Thus, technologies need to be flexible enough to accommodate these changes and remain relevant to users and the individuals who support them. From discussion sessions, we also learned that for home technology implementation, the work to support the technology (e.g., maintenance, troubleshooting) is as important as, if not more important than, the development itself. If a technology breaks and there is no one to support it, people will stop using it. Older adults need a support system to keep their technologies running. We encourage others to consider these factors when developing technologies to support older adults, incorporating community engagement and user discussions into their technology development to support successful translation into adoption.

## 5. Conclusions

This study highlighted the perceived benefits, challenges, and concerns that older adults shared regarding the adoption of home-based 24/7 sensor data collection for general health monitoring and detection of health changes. Utilizing both surveys and discussion group sessions, participants shared insights into the benefits and drawbacks of using technology to support their health and safety, highlighting the need for flexible, cost-conscious, and user-friendly systems that respect their autonomy. The findings underscore the importance of including potential users throughout the technology development process to create solutions that are both acceptable and effective for the growing aging population. Our study identified numerous physical, mental, and support challenges that need to be addressed in any technology development to facilitate adoption by this growing population segment. Configurations appropriate for a young older adult may not be sufficient or usable by that individual as they age into their 80s and beyond. Thus, technology development must be flexible and adaptive, tailored to an individual’s changing needs as they age, respecting users’ comfort with home monitoring and adapting to their preferences regarding data sharing and in-home sensor location. Future work should also focus on integrating health-related data generated from smart home monitoring into a comprehensive health monitoring program. Full community engagement and acceptance will be required throughout the technology development, testing, and deployment lifecycle to ensure the successful development of sensor technology that is age-friendly and supportive of all potential users.

## Figures and Tables

**Table 5:** Summary of discussion sessions themes and key findings.

Major Theme	Subtheme	Key Findings and Participant Concerns
Theme 1: Challenges to aging in place	Assistance Needed	- Just got discharged from the hospital. I am afraid to be alone, especially at night - Taking so many medications, I get confused - Having trouble seeing my phone to make calls - Cannot cook much anymore. Can’t stand too long, can’t lift my arms to reach stuff from high cabinets or bend down to take things out of low cabinets - Want to FaceTime with my kids, but since the internet “broke”, I have no one to help me fix it - I keep fighting with my kids. I want to stay in my home, but they are worried that I have trouble with the stairs to get to my bedroom and bathroom - My hearing is not what it used to be. Cannot hear the doorbell or people on the phone - Hard to get assistance on the phone; everything is automated. I need to speak with a person - Technology is so expensive and hard to use. I go to the library for Wi-Fi - Had to stop driving. What am I going to do now?
	Facing Isolation or Loneliness	- Recently, I became a widow and am living alone - If home alone, having a personal monitor is a great idea if you’re at risk of falling or passing out, or if you have a problem - Want to FaceTime with my kids, but since the internet “broke” I have no one to help me fix it - I have no one close by to help me - I am getting so old, all my friends are either moving away or dying - Since retiring, I am home alone during the day, if something happens, no one will know - At high risk for COVID so I stay away from things I like—movies, theater, senior center, museums, travel - Since COVID, it is harder to get together with friends and family
Theme 2: Home monitoring concerns and questions	Privacy	- I value my privacy. I don’t want people to see me from the sensor. - I would like to turn the sensors on and off when I want to - Don’t want to be monitored 24/7 - Security. Worried about hacking! - What data will be collected and from whom? Do I have to get permission from visitors before they come into my home? - Who will see my data - I am afraid of losing my independence if I am monitored at home - Don’t want data collected on my grandkids when they visit - Do not want sensors in the bedroom or bathroom. Don’t want my kids to see me naked
	Burden	- Added expense. I am on a fixed income - Too complicated to use. I am not technologically savvy - Who will support the technology? At what cost? - Don’t want to burden my family with a lot of data - Who will fix it if it breaks? - Don’t want to be overwhelmed by the data - How will I share these data with my healthcare provider? - How will these data help with monitoring my health - Afraid the data will overwhelm my healthcare provider
Theme 3: Caregiver concerns	Caregiver/ Life Balance	- We had to move because my husband could no longer go up the stairs. I lost all my friends - I need more help to identify my aunt’s problems remotely - If Mom doesn’t answer the phone when we call, we have to get in the car and drive an hour to make sure she is ok - I am so tired, I don’t sleep. I worry about my wife, who gets up at night. She has even walked outside without shoes and a coat - My parents want to stay in their home as long as possible. We have no extra room for them to move in - I cannot watch Mom 24 h a day - My dad has so many appointments. How do we balance work and going with him - Our kids live on the West Coast. What good will it do to call them if we have a problem - Still helping to address problems that arose because of COVID isolation. Zoom masked seeing physical decline
	Safety	- Wish my aunt wouldn’t leave the stove on and forget about it - My dad forgets to eat. I prepare his food, but find it still in the refrigerator days later - My mom has poor balance and falls often. She had been on the floor for hours before one came home - My mom cannot hear the house phone to answer it - My wife, who gets up at night, has even walked outside without shoes and a coat - My dad has dementia and likes to wander. We cannot always be in the same room to keep track of him. - I worry about my parents living 5 miles from the nearest neighbor - Wish there was a way to know my husband is ok at home when I have to run errands - Moved to a 55+ development to have services available

## Data Availability

Deidentified survey data and an associated codebook generated or analyzed in this study will be available from Dryad Digital Repository @ https://datadryad.org/stash (accessed on 25 November 2025) upon manuscript publication, DOI: 10.5061/dryad.p5hqbzm24. The dataset was reviewed by an honest broker prior to submission to confirm de-identification.

## Supplementary Materials

The following supporting information can be downloaded at: https://www.mdpi.com/article/10.3390/s25247413/s1, Supplementary S1: Technology Survey; Supplementary S2: Discussion session presentation and discussion questions.
