# Supplementary material for: Engaging Older Adults to Guide the Development of Passive Home Health Monitoring to Support Aging in Place
Source: Sensors (Basel). 2025 Dec 5;25(24):7413. doi: 10.3390/s25247413 (PMC12736577; doi:10.3390/s25247413)
Supplement: Supplementary file 1 [file sensors-25-07413-s001.zip › sensors-3825337-supplementary.pdf]

Supplementary S1

PARTICIPANT SURVEY

Stony Brook University researchers are developing new technologies to use at home to help keep individuals independent, safe, healthy and socially connected as they age. There are two main benefits from having this kind of technology installed at home. The technology can: 1) detect and notify family members, neighbors, health care providers and 911 when emergency events (e.g., falls) happen; and 2) detect changes in one's health (e.g., changes in heart rate, hand trembling) to help health care providers manage one's overall health and address changes. These sensors will collect daily activity data (e.g., walking, cooking, sleeping, bathing) and physiological data (e.g., heart beat and respiration rate). Analysis of these data will detect emergencies and changes in health status for early intervention. The system will preserve privacy, protecting the data from unauthorized parties with individuals deciding what data to share with whom and under what conditions.

We are asking individuals like you to provide input to help us understand your needs as you get older and how these technologies may help you remain safe and healthy in your homes. Thank you for taking the time to complete the survey.

a. **How did you hear about the study? (select all that apply)**
☐ OLLI ☐ LISVH ☐ Senior Center ☐ Religious Organization
☐ on-line search ☐ email invite ☐ friend/word of mouth ☐ other (specify: ________________)

b. **What state do you live in?** _______

c. **What city do you live in?** _________________________

SECTION 1: Can we start by learning a few things about you?

1. **What is your gender**: ☐ male ☐ female ☐ transgender ☐ other

2. **How old are you?** ☐ <50 ☐ 50-59 ☐ 60-69 ☐ 70-79 ☐ 80-89 ☐ 90+

3. **What is your highest level of education?**
☐ no high-school diploma ☐ high school graduate ☐ some college ☐ college graduate or higher

4. **Which of the following best describes your race (select all that apply)**
☐ American Indian/Alaska Native ☐ Other Pacific Islander
☐ Asian ☐ White
☐ Black/African American ☐ Other
☐ Native Hawaiian ☐ Prefer not to say

5. **Are you of Latino, Hispanic or Spanish origin?** ☐ Yes ☐ No

6. **What is your home 5-digit ZIP code?** ________________________(5-digit zip)

7. **What is your current marital status?**
☐ Never married ☐ Divorced ☐ Separated
☐ Married/living in a marriage like relationship ☐ Widowed ☐ Prefer not to say

8. **What is your current employment status?**
☐ Working full-time ☐ Disabled ☐ Unemployed or laid off
☐ Working part-time ☐ Retired ☐ Prefer not to say
☐ Keeping house or raising a family full time

9. Do you have any pets? ☐ no ☐ yes (type pet(s): ________________________________)

10. Which one of the following BEST describes your living arrangements? I live:

- ☐ by myself
- ☐ with a spouse/significant other
- ☐ with a child/other family member(s)
- ☐ with friends/roommates
- ☐ in a group home
- ☐ in an Independent Living Facility
- ☐ in an Assisted Living Facility
- ☐ Other, Specify: ________________________

11. Do you have a primary care provider?

☐ Yes ☐ No

When was your last visit to your primary care provider?

- ☐ Within the month
- ☐ 1 – 6 months ago
- ☐ 7 – 11 months ago
- ☐ 1-2 years ago
- ☐ > 2 years ago

11a. During COVID did you use telehealth visits with any of your health care providers?

☐ Yes ☐ No

How likely are you to continue to use telehealth as part of your health care?

☐ very likely ☐ somewhat likely ☐ somewhat unlikely ☐ very unlikely

12. If technologies existed to alert others when you fall or are not feeling well WITHOUT you having to make a phone call or push a button, how likely would you be to want to have this technology in your home? ☐ very likely ☐ somewhat likely ☐ somewhat unlikely ☐ very unlikely

13. Which of the following do you have access to at home (check either yes/no/don't know for each)

Technology	Yes	No	Don't know
Basic cellphone (e.g., flip phone)	☐	☐	☐
Smartphone (e.g. Apple iPhone, Samsung galaxy)	☐	☐	☐
Tablet (e.g. iPad, Samsung)	☐	☐	☐
Computer (desktop or laptop)	☐	☐	☐
Cable/satellite access (e.g., Spectrum, Verizon)	☐	☐	☐
Text messaging on your cellphone	☐	☐	☐
Video conferencing (e.g., Zoom)	☐	☐	☐
Internet access via Wi-Fi (wireless)	☐	☐	☐
Internet access via a wired connection	☐	☐	☐
Home health monitoring devices (e.g., blood pressure machine)	☐	☐	☐
Wearable health devices (e.g., Fitbit)	☐	☐	☐
Falls detection monitor	☐	☐	☐

14. How confident are you in using each of these technologies? (check one option for each question)

Technology	Very	Somewhat	Only a little	Not at all	Do not use
a. Making or receiving a call on your cellphone	☐	☐	☐	☐	☐
b. Using your tablet (e.g., iPad, Samsung)	☐	☐	☐	☐	☐
c. Using your computer (desktop or laptop)	☐	☐	☐	☐	☐
d. Using an app on your smartphone, tablet or computer	☐	☐	☐	☐	☐
e. Text messaging on your cellphone	☐	☐	☐	☐	☐
f. Video conferencing on your phone, tablet or computer	☐	☐	☐	☐	☐
g. Connecting to the internet	☐	☐	☐	☐	☐
h. Home health monitoring devices (e.g., blood pressure machine)	☐	☐	☐	☐	☐
i. Wearable health devices (e.g., Fitbit)	☐	☐	☐	☐	☐
j. Falls detection monitor	☐	☐	☐	☐	☐
k. Telehealth/telemedicine	☐	☐	☐	☐	☐

SECTION 2: Opinions about using contactless room-based sensors in your home

In the next section we would like your opinions about your comfort with having room-based contactless sensors installed in your home that have the potential to help monitor your health and wellbeing in your home. We would like to find out how comfortable you are with the data generated from these contactless sensors, and if installed in your home, with whom you may be comfortable sharing information generated from these sensors.

	Not comfortable at all. I would not want this in my home	A single sensor in some rooms is fine.	Multiple sensors in some rooms is fine.	I am comfortable having as many as you want in as many rooms as you want.
1. In general, how comfortable would you be to have contactless sensors installed in your home to monitor your health and wellbeing?	☐	☐	☐	☐
2. If you were home alone, how comfortable would you be to have contactless sensors installed in your home to monitor your health and wellbeing?	☐	☐	☐	☐
3. If you were sick, or had a health problem that requires continuous monitoring, how comfortable would you be to have contactless sensors installed in your home to monitor your health and wellbeing?	☐	☐	☐	☐

4. If you had contactless room based sensors installed in your home, with whom and under what conditions would you be comfortable sharing the information generated from these sensors?

	Health care provider (doctor, nurse, social worker)		Professional Caregiver (e.g., home health aide)		A company providing monitoring services		Family		Friends		Neighbor	
	YES	NO	YES	NO	YES	NO	YES	NO	YES	NO	YES	NO
With whom would you be comfortable sharing your sensor data on a **regular basis?**	☐	☐	☐	☐	☐	☐	☐	☐	☐	☐	☐	☐
With whom would you be comfortable sharing your sensor data **when you are sick or recovering** from an illness that may demand immediate attention?	☐	☐	☐	☐	☐	☐	☐	☐	☐	☐	☐	☐

5. In thinking about sensors as a tool to help you when you are either healthy or sick, in which locations would you be comfortable having them installed?

	**Living room**		**Bedroom**		**Bathroom**		**kitchen**	
	YES	NO	YES	NO	YES	NO	YES	NO
One sensor in select rooms when **I am healthy**	☐	☐	☐	☐	☐	☐	☐	☐
Multiple sensors in select rooms when **I am healthy**	☐	☐	☐	☐	☐	☐	☐	☐
One sensor in select rooms **when I am sick or recovering**	☐	☐	☐	☐	☐	☐	☐	☐
Multiple sensors in select rooms **when I am sick or recovering**	☐	☐	☐	☐	☐	☐	☐	☐

Supplementary S2

DISCUSSION SESSION
PRESENTATION AND QUESTIONS

Stony Brook University

Creating a Technology-based Program to Support Older Adults Aging in Place

Elinor Schoenfeld PhD

Renaissance School of Medicine

Fan Ye PhD and Erez Zadok PhD

College of Engineering and Applied Sciences

Patricia Bruckenthal PhD APRN-BC FAAN

School of Nursing

Shelley Horwitz MSW

School of Social Welfare

FAR BEYOND

Supported by a grant from the National Science Foundation Grant #1951880

© Research Foundation of SUNY

Stony Brook University

Our Research Goals

- Create a technologies-based personalized toolkit to support us as we age
  - *meet an individual's unique and changing health and social support needs*
  - *are easy to use, at a cost-conscious price*
  - *Support independence within and outside the home*
  - *Ensure privacy*
- To accomplish this, we are currently testing our technologies in the lab, followed by enrolling older adults to use these in their homes to:
  - *monitor health and detect changes in health for early intervention*
  - *have an alert system when there are health changes (e.g., falls)*
  - *support one's ability to function at home and socialize*
  - *identify the need for additional new technologies as we learn more*

FAR BEYOND

V3.1 10 13 2022

© Research Foundation of SUNY

We want to hear from you

Your help

- *We are here today to learn from you about your technology use and how the technologies that we are developing at Stony Brook can best be designed to help you and other individuals like you age in place.*
- *The information you provide today will help us to further develop our technologies with the goal of monitoring activities we do at home like eating and watching TV to help monitor your health and wellbeing.*

Your help

Your participation today will include 4 things:

1. Listen to a story about a fictitious older woman and her family who are looking for technologies to help her stay safely at home.
   - *We will give each of you an opportunity to share your thoughts about her challenges and the decisions she and her family made*
2. Listen to a short presentation that describes the contactless room-based sensors we are developing and our plans for addressing security and privacy concerns to help monitor health and wellbeing at home as we age
3. We will then give each of you an opportunity to share your thoughts on our technologies and how they may help you and/or a family member age in place
4. Lastly, we ask that you complete a brief anonymous research survey about your technology use and opinions about home installed sensors and the data they may collect.

© Research Foundation of SUNY

Your help

- *All the information we get about you will be kept private.*
  - *Your participation is voluntary.*
  - *We will be recording the discussion session today so we can review what was said at a later time, so please use first names only*
  - *Please do not write down your name or any contact information on the survey to keep it anonymous.*
- *Once you complete your survey, please turn it upside down and place in the center of the table.*
  - *We will come around and place completed surveys in this envelope. If you have any questions as you complete the survey, I will be happy to answer them.*

© Research Foundation of SUNY

Stony Brook University

Your help

- *If you are interested in participating in our research study, please take a look at the consent form in front of you. Once you have had time to review it, I will answer any questions you may have about participating in this survey.*
  - *If you do not want to read the consent form and/or are not interested in participating in today’s discussion, you are welcome to leave before we start.*

© Research Foundation of SUNY

Stony Brook University

Your help

- *We would be happy to answer any questions you may have about participating in today’s discussions before we get started.*
- *All the information we get about you will be kept private.*
  - *Your participation is voluntary.*
  - *You are welcome to leave at any time if you feel uncomfortable*

© Research Foundation of SUNY

Stony Brook University

Let’s meet Ashley and her Family

Stony Brook University

Let’s Meet Ashley and her family

Ashley is the mother of Johnson and Jonathan and grandmother of three beautiful girls Jessica, Alex, and Romania. Ashley spent her whole life working as a Nursing Assistant and loved taking care of others. She loved cooking for her neighbors and attending weekly book club meetings with her late husband Diego. Ashley was a chronic smoker but since being diagnosed with a lung disease that affects her breathing and high blood pressure. Now she only smokes when stressed. At 65 years old Ashley lost her husband of 40 years and is currently adjusting to living in her two-floor family house on her own. Ashley is independent but is struggling to remember to take her medication and follow doctors' orders. Getting up and down the stairs without becoming increasingly short of breath is now difficult for her. Her sons are both worried and insist on getting their mom a companion, someone to check in on her and remind her to take her medications. Ashley is having a hard time accepting that she isn’t as young and healthy as she used to be and insists she can still live on her own. She compromised with her sons and agreed to sign up for a neck-worn medical alert system like Life Alert to appease them.

Stony Brook University

Last December, Ashley, unfortunately, fell ill, she developed a productive cough, became very short of breath, weak, and dizzy. She couldn't get out of bed or call for help, so she just laid in bed trying to reach the medical alert button she had hung on the top of the headboard. She never wears the medical alert necklace when she is in bed. After what seemed like forever, she was able to gather enough energy to reach the necklace and press the button. The company immediately called her phone when they received the signal to make sure It wasn't a mistake, but she could not reach it. They then proceeded to contact her son and sent an ambulance to her house. Jonathan lived an hour away and Johnson was at work so they gave the police permission to break the door down to get to their mother and they would meet her at the hospital. When they arrived, she was unable to breathe, and they rushed her to the hospital. Upon arrival, Ashley was immediately put on a machine to help her breath and admitted to the intensive care units.

© Research Foundation of SUNY

Stony Brook University

Discussion Question

What do you think about Ashley's initial health monitoring plan?

© Research Foundation of SUNY

Stony Brook University

After she was discharged. Ashley acknowledged she had become far too weak to be on her own, but still refused to have someone take care of her. Her sons were even more concerned about their mother’s safety and decided to talk to her about having cameras and personal assistant devices installed in the bedroom, bathroom, living room, and kitchen, they could monitor their mother and set up reminders for her to take her medications and treatments on time. This idea did not sit well with Ashley, she was very uncomfortable about being recorded and even more so about her son’s watching her “like a fish in a bowl”. She didn’t even want a personal assistant device because she heard from her friend Janet, from the book club, that companies use these devices to listen in on your conversation and send you advertisements. She refused to have the “whole world” in her business. This left Johnson and Johnathan to look up other alternatives. They wanted a way to monitor their mother’s vital signs but did not want to impede on Ashley’s privacy. They discussed getting her a smartwatch, but this was very expensive, limited in what it can monitor, and she would have to wear it all the time for It to work.

© Research Foundation of SUNY

Stony Brook University

After careful research, Johnson and Jonathan discovered there was a device that would not only be able to monitor their mother’s vitals but would do so without her having to physically do anything and this data could be shared with the doctors and whoever else she chooses. This was a secure and safe way to guarantee that their mother's health would be constantly monitored but her privacy would be intact. This made them so happy because their mother could still be independent but get around the clock monitoring and help in case anything went wrong. Ashley was hesitant at first but after her sons explained how secure and helpful this system was, she agreed to give it a try. Ashley and her family can now have peace of mind knowing that if something went wrong, her doctors would be the first to know. She was able to keep her privacy and still get quality health care.

© Research Foundation of SUNY

Stony Brook University

Discussion Question

What do you think about her son’s actions?

How did it make you feel?

© Research Foundation of SUNY

Stony Brook University

Discussion Question

Focusing on the son’s idea to use cameras and personal assistant devices, what are your thoughts about Ashley’s security and privacy concerns?

© Research Foundation of SUNY

Stony Brook University

Anonymous Survey Completion

FAR BEYOND

Stony Brook University

Completing the survey

- *We will now distribute the anonymous survey for you to complete*
- *All the information we get about you will be kept private.*
  - *Your completion of the survey is voluntary. If you do not wish to complete the survey, you do not have to and are welcome to leave as the session is over.*
  - *Please do not write down your name or any contact information on the survey to keep it anonymous.*
- *Once you complete your survey, please turn it upside down and place in the center of the table.*
  - *We will come around and place completed surveys in this envelope. If you have any questions as you complete the survey, we will be happy to answer them.*

© Research Foundation of SUNY

Stony Brook University

Our Sensor System

FAR BEYOND

Stony Brook University

Our Radio-base Remote Non-wearable Sensing System

Vision: data-driven smart aging for older adults

- 24x7 real time sensing and AI analysis of physical activities and vital signs
- Detect emergencies (e.g., falls), predict trends, enable early intervention

Key technology: radio based, remote non-wearable sensing

- No burden to wear/charge devices
- No interference to daily activities

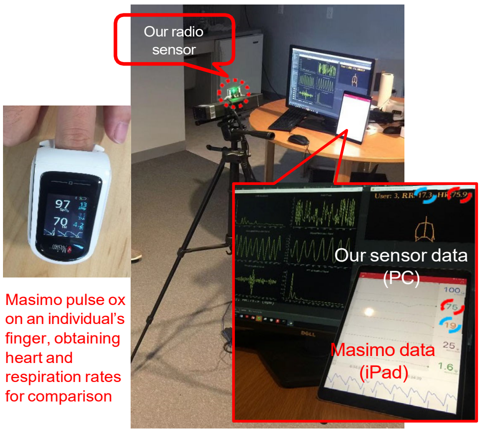

Radio sensor set up

FAR BEYOND

Stony Brook University

Real Time Measurements and Multi-person Support

- Measure vital signs in real time and compare with Masimo
- Track and differentiate multiple people

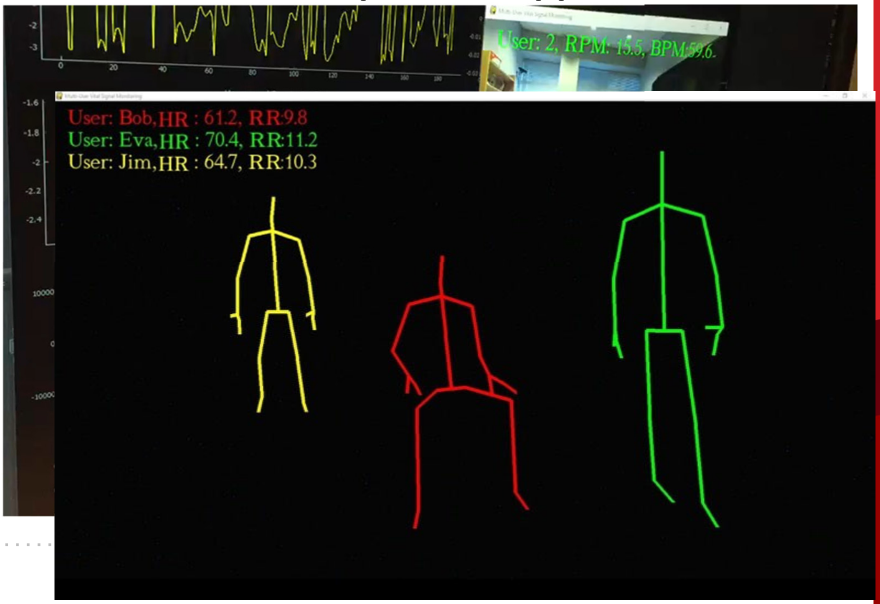

Concurrent multi-people tracking

FAR BEYOND

Stony Brook University

Comparison between Radio and Masimo Data: Overnight

FAR BEYOND

Stony Brook University

Your thoughts

If our sensor system were available for purchase, would you consider using this for yourself or a family member?

How do you think such a device would help you or a family member in everyday life?

© Research Foundation of SUNY

Stony Brook University

Security and Privacy for Aging In Place

Stony Brook University

Your opinions about sensor system security

- What kinds of security or privacy concerns would you have if our sensor system were installed in your home?
- Under what conditions would you be comfortable sharing your data with others?
  - when you are healthy, or sick?
  - when you live alone or with others?
  - in an emergency ?
  - who would you be comfortable sharing your sensor data with under any of these situations?

© Research Foundation of SUNY

Stony Brook University

Our Security & Privacy Principles

- **Use best security practices, adapt over time**
  - Log events for automated analysis
- **Users own all their data by default**
  - Users give permission for data access
- **Sensors collect the least amount of data that's still useful**
  - No image containing individual identity will be obtained
  - Data you don't have can't be leaked or stolen
- **Provide different pre-defined security policies to meet your own needs**
  - For different levels of security concerns vs. health status
  - Offer escalation and de-escalation policies (e.g., when there is a need to call 911)

© Research Foundation of SUNY

Stony Brook University

Interested in continuing your study participation?

FAR BEYOND

Stony Brook University

Future study participation

If you would like to be informed about future opportunities to participate in our study (e.g., home based sensor data collection), please complete and return to us this form with your name and contact information.

We will not share your information with any other party. We would only contact you with information about future study participation.

© Research Foundation of SUNY

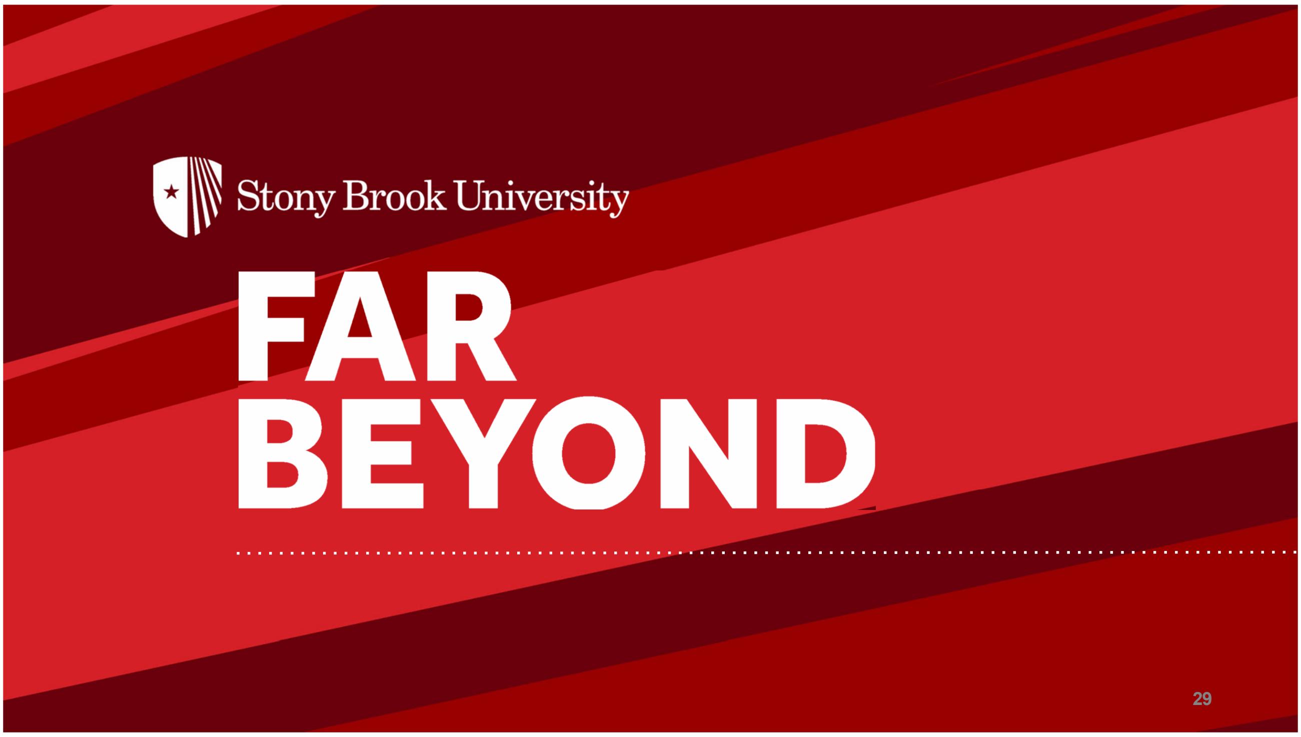
Stony Brook University
FAR
BEYOND
29
